# Trends in the Implementation of the Cyberchondria Severity Scale: Bibliometric Analysis

**DOI:** 10.2196/75003

**Published:** 2026-01-05

**Authors:** Adam C Powell, Cayetana Calderon-Smith

**Affiliations:** 1 Payer+Provider Syndicate, 20 Oakland Ave, Newton, MA, 02466, United States, 1 617-939-9168; 2 College of Population Health, Thomas Jefferson University, Philadelphia, PA, United States; 3 Therapy 2.0, Bala Cynwyd, PA, United States

**Keywords:** health anxiety, health information seeking behavior, hypochondriasis, hypochondria, Cyberchondria Severity Scale, CSS, 12-item Cyberchondria Severity Scale, CSS-12

## Abstract

**Background:**

Cyberchondria, a combination of the words “cyber” and “hypochondriasis,” is a condition that is receiving increasing attention from clinicians and researchers globally. Researchers are currently using multiple instruments to quantify it. Furthermore, the instruments have been translated into multiple languages.

**Objective:**

This study aimed to examine the extent to which researchers are measuring cyberchondria using the 33-item Cyberchondria Severity Scale (CSS) and its 12-item abbreviated version, the CSS-12. It also examined the relative use of cyberchondria instruments in different languages.

**Methods:**

PubMed and PsycInfo were searched for articles published between May 1, 2019, and December 31, 2024, featuring the term “cyberchondria” in the title. Included articles mentioned the CSS, were empirical studies, and were in English. Each article was categorized by the CSS version, publication year, and language of instrument implementation. Fisher exact tests were used to assess associations, and the Spearman rank correlation coefficient was used to evaluate trend monotonicity.

**Results:**

Among the 117 articles included in the analysis, 42 (35.9%) used the CSS, 38 (32.5%) used the CSS-12, and the remaining 37 (31.6%) used unknown or modified versions. Although CSS-12 use began with its introduction in 2019, there was no significant association between publication year and instrument choice (*P*=.84). Unadjusted analysis found that the relationship between year and the percentage of articles using the CSS-12 showed a statistically significant monotonic trend (ρ=0.89; *P*=.02). This finding was not significant after applying a Bonferroni correction. However, there was a significant association between the language of the instrument and the CSS version used (*P*<.001).

**Conclusions:**

From 2019 to 2024, both the CSS and CSS-12 continued to be used. The CSS-12 offers benefits such as brevity and the removal of reverse-keyed items, while the original CSS remains useful for studies that require the mistrust of medical professionals subscale. The significant association between language and instrument choice suggests that cultural and linguistic factors impact selection, and instrument choice should be guided by the study’s objectives and the constructs of interest.

## Introduction

### Definition and Current Measurement Tools

Cyberchondria is a portmanteau of the words “cyber” and “hypochondriasis.” Its measurement was first formalized through the development of the Cyberchondria Severity Scale (CSS) in 2014 [1]. In its original format, the CSS consists of a 33-item questionnaire, grouped into 5 subscales, some of which identify behaviors (ie, compulsiveness, excessiveness, and reassurance seeking) or mental states (ie, distress). An additional subscale, mistrust of medical professionals, has the potential to be problematic, as it may measure a construct that is *different from, but related to,* the other 4 cyberchondria subscales [2].

In response to both the length of the original, long-form CSS and the potential issues surrounding the mistrust of medical professionals subscale, an abbreviated version called the CSS-12 was developed in 2019 [3]. The CSS-12 consists of a 12-item questionnaire containing questions drawn from the original version; however, it does not include any items related to the mistrust of medical professionals. The creators of the original CSS were involved in the development and validation of CSS-12 and thus have implicitly endorsed it.

Since their creation, the CSS and CSS-12 have been used in numerous studies and have become de facto standards for the measurement of cyberchondria. A potential overreliance on the CSS is acknowledged in the literature [4]. Furthermore, the instruments have been translated into other languages and have been extensively used in adapted forms. On this note, in 2016, a German team created the 15-question German version of the instrument, dubbed the CSS-15 [5]. Additional novel instruments have been developed, some of which include the aforementioned mistrust of medical professionals construct [6,7].

### Study Aims

There is widespread use of the CSS and CSS-12 and a lack of research comparing their relative use. To address this lacuna, this study aims to provide future researchers with greater understanding of the extent to which each version is used and the degree to which each version is being used in languages other than English. It additionally aims to contribute to the discussion of the various contexts in which inclusion of the mistrust of medical professionals subscale is helpful. To achieve these aims, we conducted a review of the literature to determine the relative frequency with which the CSS and CSS-12 were used and the languages in which they were used. While conducting this review, situations in which noncanonical forms of the questionnaire were used were noted.

## Methods

### Ethical Considerations

Ethics approval and informed consent were not applicable because this study examined the published literature, rather than human participants.

### Search Strategy and Sample Selection

In September 2025, PubMed and PsycInfo were searched for all potentially relevant articles published between May 1, 2019, the date of publication of the article defining the CSS-12, and December 31, 2024, the last day of the most recent calendar year. PubMed is a free tool that searches the archive of biomedical and life sciences journal literature maintained by the United States National Library of Medicine. It may be most accessible to clinical practitioners without institutional access to paywalled sources. PsycInfo is a database of articles administered by the American Psychological Association. Articles likely to be about cyberchondria were initially identified by searching for peer-reviewed, published articles with “cyberchondria” in the title. A pool of articles to evaluate was created by removing the duplicates found by both sources. Articles were excluded if they were replies, corrigenda, letters to the editor, letters from the editor, or not actually published during the search period. Further exclusions were made for articles that were not in English, were reviews, contained conceptual analysis, or did not measure cyberchondria.

### Measurement

Each article was reviewed to determine whether it used the original 33-question CSS, the CSS-12, another form of the CSS (eg, the CSS-15 or an author-derived version), or an unknown version. Culturally equivalent translations of the CSS or CSS-12 from English into another language were classified as being the instrument that was translated. The process used to determine the version of the scale used is described in Multimedia Appendix 1. Two variables were created to capture the language of the instrument: 1 variable that categorized studies as having used an instrument with an “unspecified” language if it was not explicitly stated and 1 variable that attempted to infer the language of the instrument used in studies based upon the context in which they were conducted. The process used to ascertain the language used in an article is described in Multimedia Appendix 2.

Articles were additionally classified by year of publication and by the language in which the instrument was implemented. While only English-language articles were considered, articles were written by authorship teams from various nations and, in many cases, reported on empirical research that was not conducted in English. Studies conducted in English-speaking countries were assumed to have used an English version of the instrument, unless explicitly stated otherwise. This assumption was made, as the original implementations of the CSS and CSS-12 were in English.

### Analysis

For each year, 2019 to 2024, the number of articles using the CSS, the CSS-12, and other variations of the CSS was determined by reviewing the contents of the articles meeting the sample selection criteria, and results were recorded in a table. If the version of the CSS used could not be determined, it was classified as “unknown.” Fisher exact tests were used to assess the relationship between year and the type of CSS instrument used, considering both the totality of the articles and a subset using only the CSS or CSS-12. Spearman rank correlation coefficient was calculated to determine whether there was a trend in the percentage of cyberchondria articles using the CSS-12 that was monotonic. The percentage of cyberchondria articles using the CSS-12 was then plotted by year using a scatterplot.

The sample was examined to determine the language used to assess cyberchondria in each study considered. For each language found in the sample, the number of studies using the CSS, the CSS-12, and author-derived variations of the CSS was determined. Fisher exact tests were run to assess whether a significant association existed between language and CSS implementation used, again considering both the totality of the articles and the subset using only the CSS or CSS-12.

## Results

**Figure 1.**
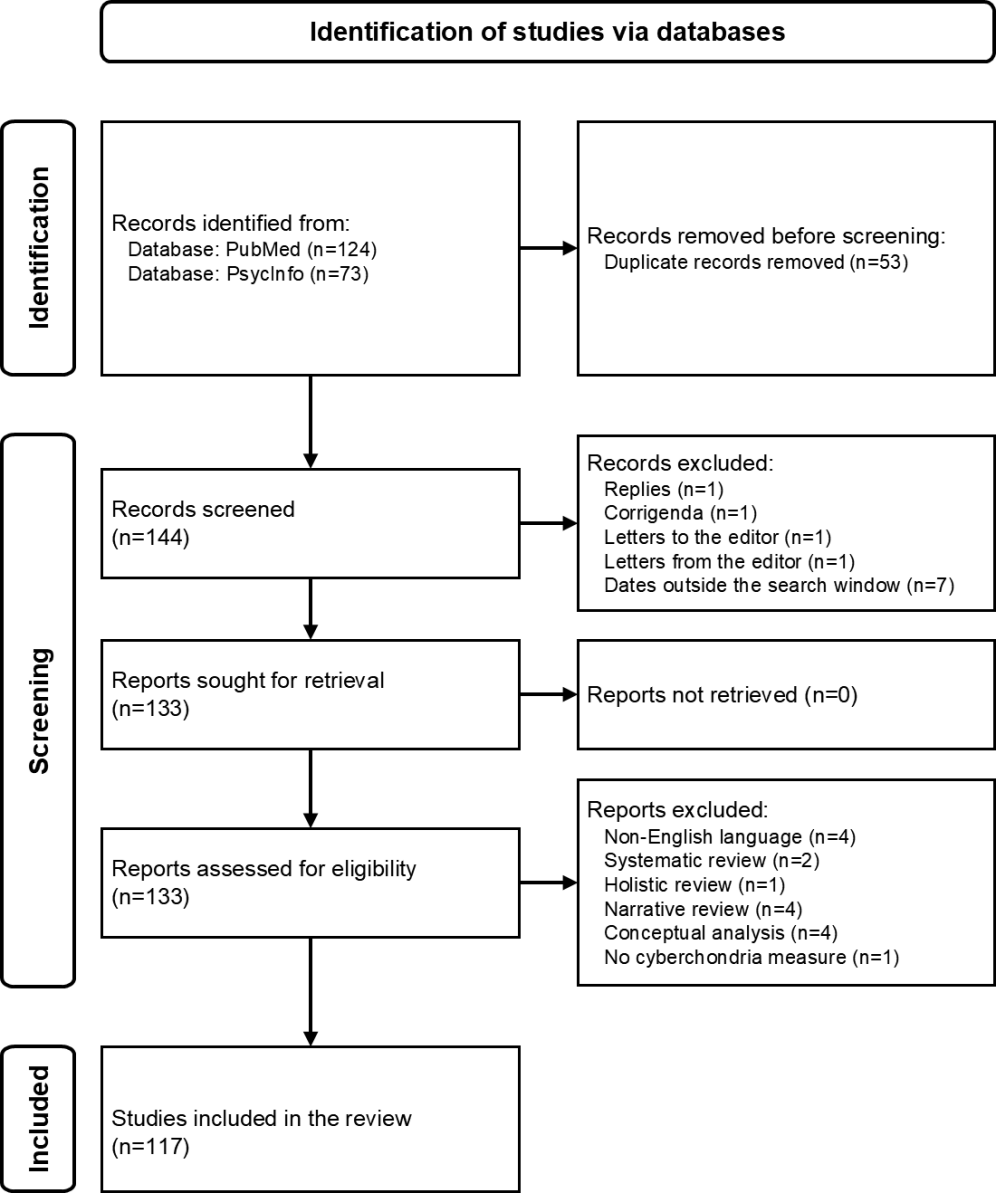
PRISMA (Preferred Reporting Items for Systematic Reviews and Meta-Analyses) diagram depicting the sample creation process.

Searching PubMed yielded 124 articles, and searching PsycInfo yielded 73 articles. Of these 197 articles, 144 (73.1%) were unique. The exclusion criteria were applied as shown in the PRISMA (Preferred Reporting Items for Systematic Reviews and Meta-Analyses) diagram (Figure 1), leading to 117 (59.4%) studies being included in the review. Of these 117 articles, 42 (35.9%) used the CSS, 38 (32.5%) used the CSS-12, 36 (30.8%) used other instruments, and 1 (0.9%) used an unknown instrument.

As shown in Table 1, while the CSS-12 was introduced in 2019, it took some time for it to gain widespread use after its initial introduction [3]. Only 11% (1/9) of the articles used it in 2020, and only 36% (5/14) of the articles used it in 2021. A Fisher exact test did not identify a significant association between the year of publication and the instrument used (*P*=.84). When a Fisher exact test was run considering only studies that used the CSS or CSS-12 (excluding studies using instruments classified as other and unknown), there was still no significant relationship between the year of publication and the instrument used (*P*=.54). Spearman rank correlation coefficient showed a statistically significant monotonic relationship between the year of publication and the proportion of studies using the CSS-12 (ρ=0.89; *P*=.02). The year in which the greatest proportion of the studies used the CSS-12 was 2024, when 39% (9/23) of the studies used the instrument.

**Table 1.** Instrument use by year, 2019 to 2024.

	CSS^a^, n (%)	CSS-12^b^, n (%)	Other, n (%)	Unknown, n (%)
2019 (n=6)	2 (33.3) [8,9]	1 (16.7) [3]	3 (50) [7,10,11]	0 (0)
2020 (n=9)	5 (55.6) [12-16]	1 (11.1) [17]	3 (33.3) [18-20]	0 (0)
2021 (n=14)	6 (42.9) [21-26]	5 (35.7) [27-31]	3 (21.4) [32-34]	0 (0)
2022 (n=39)	15 (38.5) [35-49]	12 (30.8) [50-61]	11 (28.2) [62-72]	1 (2.6) [73]
2023 (n=26)	6 (23.1) [74-79]	10 (38.5) [80-89]	10 (38.5) [90-99]	0 (0)
2024 (n=23)	8 (34.8) [100-107]	9 (39.1) [108-116]	6 (26.1) [6,117-121]	0 (0)
Grand total (n=117)	42 (35.9)	38 (32.5)	36 (30.8)	1 (0.9)

a CSS: Cyberchondria Severity Scale.

b CSS-12: 12-item Cyberchondria Severity Scale.

**Figure 2.**
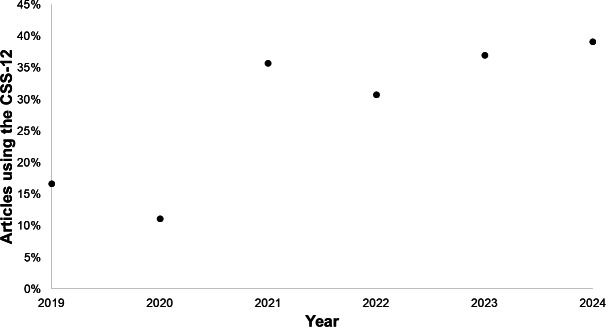
Percentage of cyberchondria articles using the 12-item Cyberchondria Severity Scale (CSS-12) by year.

As shown in the scatterplot presented in Figure 2, the only cyberchondria article published in 2019 mentioning the CSS-12 was the article that defined the instrument [3]. Use of the CSS-12 exceeded use of the CSS in 2023 and 2024, but studies using the CSS-12 did not account for the majority of studies due to the various other versions of the instrument that were used.

As shown in Table 2, among the articles that explicitly stated the language that was used, the CSS saw the greatest adoption in articles that implemented it in Turkish (14/42, 33%), and the CSS-12 saw the greatest adoption in articles that implemented it in Chinese (6/38, 16%) or Turkish (6/38, 16%). The languages for which there were more articles written using the CSS-12 than the CSS were Arabic (4/5, 80%), Chinese (6/9, 67%), Persian (3/4, 75%), Russian (1/1, 100%), Spanish (2/2, 100%), and Serbian (1/2, 50%). A Fisher exact test identified a significant association between the language in which an article implemented its cyberchondria measurement and the instrument used (*P*<.001). When articles using an instrument other than the CSS or CSS-12 were excluded from the analysis, a Fisher exact test likewise identified a significant association between the language in which an article implemented its cyberchondria measurement and the instrument used (*P*=.03).

**Table 2.** Instrument use by language (ambiguous cases classified as “unspecified”).

Language	CSS^a^, n (%)	CSS-12^b^, n (%)	Other, n (%)	Unknown, n (%)
Arabic (n=5)	0 (0)	4 (80) [27,80-82]	1 (20) [117]	0 (0)
Chinese (n=9)	2 (22.2) [35,74]	6 (66.7) [28,50,83,108-110]	1 (11.1) [90]	0 (0)
Croatian (n=6)	1 (16.7) [100]	0 (0)	5 (83.3) [7,18,91,92,118]	0 (0)
English (n=18)	7 (38.9) [8,9,12,13,21,22,36]	5 (27.8) [3,84,111-113]	5 (27.8) [10,32,62,93,119]	1 (5.6) [73]
French (n=1)	0 (0)	1 (100) [85]	0 (0)	0 (0)
German (n=3)	1 (33.3) [101]	0 (0)	2 (66.7) [19,63]	0 (0)
Indonesian (n=1)	0 (0)	0 (0)	1 (100) [64]	0 (0)
Italian (n=6)	3 (50) [14,37,38]	3 (50) [29,51,52]	0 (0)	0 (0)
Korean (n=1)	0 (0)	0 (0)	1 (100) [94]	0 (0)
Persian (n=4)	1 (25) [75]	3 (75) [17,30,53]	0 (0)	0 (0)
Polish (n=8)	7 (87.5) [15,23,24,39,102-104]	1 (12.5) [54]	0 (0)	0 (0)
Portuguese (n=1)	1 (100) [40]	0 (0)	0 (0)	0 (0)
Russian (n=1)	0 (0)	1 (100) [55]	0 (0)	0 (0)
Serbian (n=2)	0 (0.0)	1 (50.0) [56]	1 (50.0) [95]	0 (0.0)
Spanish (n=2)	0 (0)	2 (100) [57,86]	0 (0)	0 (0)
Turkish (n=24)	14 (58.3) [25,41-46,76-79,105-107]	6 (25) [31,58-60,87,114]	4 (16.7) [33,65,66,96]	0 (0)
Urdu (n=1)	0 (0)	0 (0)	1 (100) [99]	0 (0)
Unspecified (n=24)	5 (20.8) [16,26,47-49]	5 (20.8) [61,88,89,115,116]	14 (58.3) [6,11,20,34,67-72,97,98,120,121]	0 (0)
Grand total (n=117)	42 (35.9)	38 (32.5)	36 (30.8)	1 (0.9)

a CSS: Cyberchondria Severity Scale.

b CSS-12: 12-item Cyberchondria Severity Scale.

In Table 2, of the 117 articles, the language of the instrument was unspecified in 24 (20.5%), as no explicit statement was provided. However, because the language can often be inferred from the national context in which the study was conducted, Table 3 reports scale use by language, incorporating both explicitly stated and inferred languages. In this revised analysis, studies implementing the traditional CSS in Turkish were most common (15/42, 36% articles), whereas among studies using the CSS-12, those implementing it in English (7/38, 18%) or Turkish predominated (7/38, 18%).

**Table 3.** Instrument use by language (languages inferred for ambiguous cases).

Language	CSS^a^, n (%)	CSS-12^b^, n (%)	Other, n (%)	Unknown, n (%)
Arabic (n=6)	0 (0)	4 (66.7) [27,80-82]	2 (33.3) [67,117]	0 (0)
Chinese (n=16)	2 (12.5) [35,74]	6 (37.5) [28,50,83,108-110]	8 (50) [6,34,68,71,72,90,120,121]	0 (0)
Croatian (n=6)	1 (16.7) [100]	0 (0)	5 (83.3) [7,18,91,92,118]	0 (0)
English (n=28)	9 (32.1) [8,9,12,13,21,22,36,47,48]	7 (25) [3,84,111-113,115,116]	11 (39.3) [10,11,20,32,62,69,70,93,97,98,119]	1 (3.6) [73]
French (n=1)	0 (0)	1 (100) [85]	0 (0)	0 (0)
German (n=3)	1 (33.3) [101]	0 (0)	2 (66.7) [19,63]	0 (0)
Indonesian (n=1)	0 (0)	0 (0)	1 (100) [64]	0 (0)
Italian (n=6)	3 (50) [14,37,38]	3 (50) [29,51,52]	0 (0)	0 (0)
Korean (n=1)	0 (0)	0 (0)	1 (100) [94]	0 (0)
Persian (n=5)	1 (20) [75]	4 (80) [17,30,53,61]	0 (0)	0 (0)
Polish (n=9)	7 (77.8) [15,23,24,39,102-104]	2 (22.2) [54,88]	0 (0)	0 (0)
Portuguese (n=1)	1 (100) [40]	0 (0)	0 (0)	0 (0)
Romanian (n=2)	2 (100) [16,26]	0 (0)	0 (0)	0 (0)
Russian (n=1)	0 (0)	1 (100) [55]	0 (0)	0 (0)
Serbian (n=2)	0 (0)	1 (50) [56]	1 (50) [95]	0 (0)
Spanish (n=2)	0 (0)	2 (100) [57,86]	0 (0)	0 (0)
Turkish (n=26)	15 (57.7) [25,41-46,49,76-79,105-107]	7 (26.9) [31,58-60,87,89,114]	4 (15.4) [33,65,66,96]	0 (0)
Urdu (n=1)	0 (0)	0 (0)	1 (100) [99]	0 (0)
Grand total (n=117)	42 (35.9)	38 (32.5)	36 (30.8)	1 (0.9)

a CSS: Cyberchondria Severity Scale.

b CSS-12: 12-item Cyberchondria Severity Scale.

As was the case in which languages were not inferred, a Fisher exact test found a significant association between the language in which an article implemented its cyberchondria measurement and the instrument used (*P*<.001); this association remained significant (*P*=.03) when articles using an instrument other than the CSS or CSS-12 were excluded.

## Discussion

### Adoption Trends

From the results in Table 1, it appears that the CSS-12 [3] had not completely replaced the CSS [1] in 2024. Given that there is no financial cost to switching instruments, it would be expected that the CSS-12 would completely replace the CSS over time if the 2 were perfect substitutes. This would be expected, as the CSS-12 is less time intensive to administer and is potentially less confusing for respondents due to its lack of reverse-keyed questions (eg, those measuring mistrust of medical professionals). The CSS-12’s shorter length is potentially beneficial for both completion rates and the cost of administration. The main barriers to adoption of the CSS-12 in a study are researcher awareness and development of the study design after gaining awareness of the CSS-12. That said, the correlation between year and the proportion of cyberchondria studies using the CSS-12 achieved significance (ρ=0.89; *P*=.02), and it appears that there was a monotonic relationship trending toward greater use of the CSS-12 over time.

### Measurement of the Mistrust of Medical Professionals

Given that the CSS-12 had been available for more than 4 years by the start of 2024, the fact that out of 23 studies, 8 (34.8%) used the original CSS in 2024 suggests that the CSS-12 may not be a perfect substitute. One key difference between the CSS and CSS-12 is that the CSS contains a subscale related to the mistrust of medical professionals, whereas the CSS-12 does not. Furthermore, this omission in the CSS-12 also makes it less suitable as an instrument for the study of the relationship between the mistrust of medical professionals and cyberchondria or other health issues, such as health anxiety [122].

Further research needs to be conducted to determine whether mistrust of medical professionals is a subconstruct related to, but distinct from, cyberchondria [2,13,38,123]. Concern over it being a distinct construct initially prompted its removal [3]. Some authors have opted to use the CSS without the reverse-keyed mistrust of medical professionals questions, citing concerns with the 5-factor structure of the CSS [8,24,36,47]. However, as the cyclical, reinforcing role of problematic digital information searches has been proposed to be a focal feature of cyberchondria presentations [124], barriers to accessing information from medical professionals constitute a concern of significant relevance. A lack of trust in health care providers broadly identifies a potential barrier to the access, use, and provision of care.

Measuring mistrust of medical professionals is relevant in public health and clinical care settings. Globally, most people do not trust medical professionals. The Wellcome Global Monitor 2020, a survey of more than 119,000 people residing in 113 countries, found that only 45% of people trust physicians and nurses in their country [125]. Measuring mistrust of medical professionals is increasingly important due to the erosion of trust that occurred during the COVID-19 pandemic. A repeated survey of Americans found that the proportion of adults who reported “a lot” of trust for physicians and hospitals declined from 71.5% in April 2020 to 40.1% in January 2024 [126]. These data suggest that the percentage of Americans with some doubts about the trustworthiness of medical professionals became the majority over this period. Furthermore, the study did not find signs that trust was rebounding. As mistrust of medical professionals becomes more common, it may be worth further exploring the nature of its association with cyberchondria, or its potential role as a control variable [127]. As these applications can only be performed with the original, long-form CSS, they provide a potential source of relevance for the measure going forward.

Moreover, measuring mistrust is important because cyberchondria can harm health care relationships between health care providers and patients in primary care settings [128,129] and may lead to “doctor shopping.” Despite its impact on use, the degree to which mistrust impacts health care utilization has been underexplored [103]. Furthermore, health care providers may experience patients with cyberchondria as difficult to treat [128], which could lead to increased clinician burnout or stress. Outside of primary care settings, specifically within psychotherapy, strong care relationships are associated with positive outcomes [130]. This suggests that measuring and managing mistrust may alert health care providers to patients who may require additional communication or support. Additionally, across the reviewed literature, the importance of successful health care provider and patient communication is often referenced [49,51,54,73], and additional literature specifically mentions the importance of care alliances [131]. Consequently, identifying these patients may combat potential clinician burnout or stress and could arguably support successful care outcomes across medical and psychotherapeutic settings.

Infodemic-related concerns are also linked to cyberchondria [132] and are referenced in the reviewed literature [101,111,118]. This factor places strain on health systems [133] and may be of special relevance to the mistrust of medical professionals construct, as patients may encounter information online that contradicts their health care providers’ recommendations. Digital literacy, for example, has been suggested as a supportive generalist cyberchondria intervention [116] and was included in the sole intervention identified in our review [14]. That said, higher digital literacy is also associated with higher cyberchondria scores, and the relationship may be mediated, moderated, or associated with other constructs [99,100,110,121].

### Social Contagion in Instrument Selection

If an author uses an instrument while working on 1 study, or sees an instrument cited in a study written by someone within their professional or social network, they may be more likely to use it. Social contagion has been demonstrated in other clinical contexts. Specifically, it has been shown that there was social contagion in surgeons’ adoption of perioperative advanced imaging when performing surgeries for the treatment of breast cancer. Patients treated by surgeons whose peers had the highest rates of imaging use were more likely to receive imaging than patients treated by surgeons whose peers had lower rates of use [134]. Likewise, social contagion may impact a researcher’s desire to pursue a study on a topic such as cyberchondria.

In 2016, a German research group produced a 15-item version of the CSS in the German language [5]. It has been noted that some items loaded on different factors in the German implementation of the CSS than in the original version, creating a fundamental difference [5,14]. The German 15-item version of the CSS was translated into English and used by several India-based researchers [10,11,70]. This repeated use of a nonstandard version of the CSS may illustrate social contagion, especially because the modified instrument was reused in a different country and language than the one in which it originated. Additionally, both canonical versions of the scale are designed for English language use and therefore could be easier to deploy in a country that uses English as an official working language. Furthermore, the loading of items onto different factors in the German 15-item version of the CSS has the potential to reduce the comparability of studies based upon this implementation of the CSS versus other versions. Social contagion and ease of implementation may also explain why researchers using one language, such as Turkish, favor the CSS, while those using another, such as Chinese, favor the CSS-12. Further research is needed to assess the impact of social contagion on instrument selection.

### Issues Related to Localization

There are both benefits and drawbacks to the localization of the CSS and CSS-12 into various languages. Providing patients with written materials in their native languages has been shown to improve comprehension [135]. However, translations of an instrument into a language may vary across researchers, leading to inconsistency in implementation, even when the same underlying instrument is used. For instance, some English medical terms have been shown to have multiple Arabic equivalents, potentially leading translations to differ [136]. Furthermore, the somatic features of depression have been shown to vary across cultures, suggesting that even standardized medical terms may be conceptualized and experienced differently by people in different contexts [137]. The developers of the Chinese CSS stated that cultural factors may influence both the presence of and responses to cyberchondria-like behaviors. Within a Chinese context, both linguistic and cultural factors influence instrument translation; “excessiveness” is a noteworthy example, as the authors explain that simply choosing to see a physician may be seen as excessive in China [74]. Finally, as the original CSS contains reverse-keyed questions and the CSS-12 does not, the CSS-12 may confer additional clarity or interpretation advantages when translated.

### Multiple Hypothesis Testing

As the study used multiple hypothesis tests, it is possible that some statistically significant findings were false positives. The analysis included 6 Fisher exact tests and 1 Spearman rank correlation coefficient, for a total of 7 hypothesis tests. If the desired significance threshold is *α*=.05, then the Bonferroni correction implies that findings would remain significant only if the *P* value was <.007.

While Spearman rank correlation coefficient showed a statistically significant (*P*=.02) monotonic relationship between the year of publication and the proportion of studies using the CSS-12 before the Bonferroni correction was applied, the relationship was not statistically significant after considering the Bonferroni correction. The Fisher exact tests assessing the association between use of any cyberchondria scale and language of implementation, using the data in Tables 2 and 3, were all significant at the *P*<.001 level. These results therefore remained statistically significant after application of the Bonferroni correction. However, when the analyses were restricted to articles that used only the CSS or CSS-12, the Fisher exact tests for Tables 2 and 3 each yielded *P*=.03, which did not meet the Bonferroni-adjusted significance threshold.

### Limitations

While this analysis captured the articles indexed by PubMed and PsycInfo, some relevant articles not included in these databases may have been missed. Likewise, there is often a body of gray literature consisting of unpublished manuscripts that are not publicly available due to their lack of significant findings, the direction of their findings, or abandonment by their authors. Therefore, while the findings do not necessarily represent all research conducted using the CSS, they do reflect the research accessible through 2 commonly used search tools, PubMed and PsycInfo.

### Conclusions

This study examined how often the CSS and CSS-12 have been used in the literature, the languages in which they have been implemented, and the contexts in which each version may be preferable. From 2019 to 2024, both instruments continued to be used. Although the increasing adoption of the CSS-12 over time showed an unadjusted statistically significant monotonic trend (*P*=.02), this association did not remain significant after Bonferroni correction for multiple comparisons. The CSS-12 offers advantages such as brevity and the removal of reverse-keyed items, while the original CSS remains useful for studies that require the mistrust of medical professionals subscale. Researchers selecting an instrument should consider the benefits of shorter administration and improved clarity alongside the need to measure constructs unique to the full CSS, as well as the availability and quality of translations into the target population’s language. Instrument choice should be guided by the study’s objectives, the constructs of interest, and the cultural and linguistic context. Further research is needed to determine the interchangeability of adapted and translated versions with the original 33-item English CSS.

## Supplementary material

10.2196/75003 Multimedia Appendix 1 Process used to assign a scale to an article.

10.2196/75003 Multimedia Appendix 2 Process used to assign a language to an article.

## Abbreviations

CSS: Cyberchondria Severity Scale
CSS-12: 12-item Cyberchondria Severity Scale
PRISMA: Preferred Reporting Items for Systematic Reviews and Meta-Analyses
