# Supplementary material for: Trends in the Implementation of the Cyberchondria Severity Scale: Bibliometric Analysis
Source: JMIR Ment Health. 2026 Jan 5;13:e75003. doi: 10.2196/75003 (PMC12768399; doi:10.2196/75003)
Supplement: Multimedia Appendix 1 [file mental-v13-e75003-s001.pdf]

Appendix A1. Scale Coding Process

The following process was used to assign a scale to an article:

- If authors state they use the CSS or the CSS-12, without revisions to the original scale, we code it as stated and stop.
- If the authors use the CSS or the CSS-12 in addition to a non-standard form of measurement, we code it as the standard version and stop.
- If authors state they use the CSS without the Mistrust of Medical Professionals construct, we code the scale as the CSS, as subsequent literature finds this construct measures a related but distinct factor and stop.
- If authors translate the CSS or the CSS-12 to another language and the authors state the scale maintains cultural or operational fidelity with the seminal scales despite alteration of the original questions, we code the scale as the CSS or the CSS-12 and stop.
- If authors alter the original CSS or CSS-12 by removing questions not included within the Mistrust of Medical Professionals construct, we code the scale as 'Other', as each unique question captures a feature of cyberchondria as defined by the authors of both the CSS and the CSS-12 and stop.
- If the authors state their use of a scale but subsequently describe the scale using different characteristics of another scale, (e.g., describing the CSS as a 15-item measure), we code the scale as 'Other', unless the complete scale items can be verified, and stop.
- If authors state they use a different cyberchondria scale, (i.e., the SCS or another established scale), we code the scale as 'Other' and stop.
- If the authors create a custom scale derived in part from the CSS, the CSS-12, or another cyberchondria scale, we code the scale as 'Other' and stop.
- If the authors do not reference a scale, we code the scale as 'Unknown' and stop.
