# Supplementary material for: Trends in the Implementation of the Cyberchondria Severity Scale: Bibliometric Analysis
Source: JMIR Ment Health. 2026 Jan 5;13:e75003. doi: 10.2196/75003 (PMC12768399; doi:10.2196/75003)
Supplement: Multimedia Appendix 2 [file mental-v13-e75003-s002.pdf]

Appendix A2. Language Coding Process

The following process was used to assign a language to an article:

- If the authors state a language when describing (a) the study population, (b) the language used within the chosen cyberchondria scale, or (c) language fluency as criteria for including the study's sample population, we code cyberchondria scales with the corresponding language and stop.
- If the authors do not state a language, but the scale was originally developed for use within that population's primary language, we code the scale's language to correspond with the stated language and stop.
- If the authors' study is conducted within a country where English is the sole primary language and CSS or CSS-12 are used yet language is not stated, we code the scale as 'English', as the original CSS and CSS-12 scales were developed in English and stop.
- If authors work within a country where English is a working language and the CSS or CSS-12 are used yet language is unstated, we code the scale as 'Suspected Language: English' unless otherwise specified, as the original CSS and CSS-12 scales were developed in English and stop.
- If the authors work within a country where English is a working language and an alternate scale originally developed for use in another language (e.g., the SCS) is used — yet the authors do not mention translation of the scale to the country's working language(s) — we code the scale as 'Suspected Language: English', as it is unlikely the sample would have a working knowledge of the scale's original language of origin and stop.
- If authors explicitly mention the language of another scale used within the study but not the cyberchondria scale, we code cyberchondria scales as 'Unspecified' and code the suspected language to correspond with the primary language of the country and stop.
- If authors do not mention a language and the working language of the country is not English, we code the scale as 'Unspecified' and code the suspected language as the working or primary language of the country and stop.
